# The distribution of the anti-HIV drug, tenofovir (PMPA), into the brain, CSF and choroid plexuses

**DOI:** 10.1186/1743-8454-3-1

**Published:** 2006-01-03

**Authors:** Christy Anthonypillai, Julie E Gibbs, Sarah A Thomas

**Affiliations:** 1 King's College London, Wolfson Centre for Age-related Diseases, Guy's Campus, Hodgkin Building, London SE1 1UL, UK; 2 Clinical Neurosciences (Epilepsy Group), St Georges University of London, Cranmer Terrace, London SW17 0RE, UK

## Abstract

**Background:**

Tenofovir disoproxil fumarate, a prodrug of the nucleotide reverse transcriptase inhibitor, tenofovir (9-[9(R)-2-(phosphonomethoxy)propyl]adenine; PMPA), was recently approved for use in the combination therapy of human immunodeficiency virus (HIV)-1 infection. This study was undertaken to understand PMPA distribution to the virus sanctuary sites located in the brain, CSF and choroid plexuses and to clarify its possible role in reducing the neurological problems associated with HIV infection.

**Methods:**

The methods used included an established bilateral carotid artery perfusion of [^3^H]PMPA and a vascular marker, D-[^14^C]mannitol, in anaesthetised guinea-pigs followed by scintillation counting, HPLC and capillary depletion analyses. Movement of [^3^H]PMPA into the brain, cisternal CSF and lateral ventricle choroid plexus was also examined in the absence and presence of additional anti-HIV drugs and a transport inhibitor. Control and test groups were compared by ANOVA or Student's *t*-test, as appropriate.

**Results:**

The distribution of [^3^H]PMPA in the cerebrum, cerebellum, pituitary gland and cerebral capillary endothelial cells was not significantly different to that measured for D-[^14^C]mannitol. However, [^3^H]PMPA accumulation was significantly higher than that of D-[^14^C]mannitol in the choroid plexus and CSF. Further experiments revealed no cross-competition for transport of [^3^H]PMPA by probenecid, a non-specific inhibitor of organic anion transport, or the nucleoside reverse transcriptase inhibitors into any of the CNS regions studied. The octanol-saline partition coefficient measurement for [^3^H]PMPA was 0.0134 ± 0.00003, which is higher that the 0.002 ± 0.0004 measured for D-[^14^C]mannitol in an earlier study.

**Conclusion:**

There is negligible transport of [^3^H]PMPA across the blood-brain barrier, but it can cross the blood-CSF barrier. This is a reflection of the differing physiological and functional characteristics of the blood-CNS interfaces. Self- and cross-inhibition studies did not suggest the involvement of a transport system in the CNS distribution of this drug. However, the ability of PMPA to accumulate in the choroid plexus tissue, but not the cerebral capillary endothelial cells, and the hydrophilic nature of PMPA, does point to the possibility of a transporter at the level of the choroid plexus. PMPA that has crossed the choroid plexus and is in the CSF could treat HIV-infected perivascular and meningeal macrophages, but it is unlikely to reach the infected microglia of deep brain sites.

## Background

**Figure 1:**
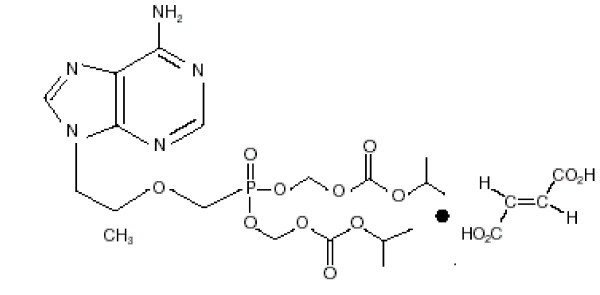
Structure of tenofovir disoproxil fumarate.

Tenofovir (9-[9(R)-2-(phosphonomethoxy)propyl]adenine; PMPA) is an acyclic nucleotide analogue with potent *in vitro *and *in vivo *antiretroviral activity. Despite its demonstrated antiviral potency, tenofovir has limited oral bioavailability in animals, presumably resulting from the presence of two negative charges on the phosphonyl group [1]. These observations have led to the design of a novel oral prodrug of PMPA, tenofovir disoproxil fumarate (Viread; Gilead Sciences Inc. and Figure 1). The addition of the disoproxil moiety aids oral absorption and once in the circulation, tenofovir is rapidly liberated and can be absorbed into cells where cellular enzymes directly produce the active metabolite, tenofovir diphosphate. Tenofovir diphosphate competitively inhibits human immunodeficiency virus (HIV) reverse transcription and causes chain termination of the nascent viral cDNA. Several clinical trials have successfully demonstrated the efficacy and favorable safety profile of tenofovir disoproxil fumarate in HIV-infected patients [2-5] and it is approved for use in the treatment of HIV-1 infection as a once-daily drug in combination with other antiretroviral regimens [6].

Highly active anti-retroviral therapy (HAART), where three or more anti-HIV drugs are used in parallel, dramatically reduces the mortality and morbidity associated with HIV infection, and is the recommended treatment strategy for HIV infection [7]. However, even with treatment, total eradication of HIV-1 appears impossible [8,9]. This is due to the presence of virus in host cellular and anatomical reservoirs that are inaccessible to HAART, and consequently are a source of viral rebound to the plasma if therapy is discontinued or inadequate [10-12]. A number of different cellular reservoirs of HIV have been identified, these include: CD4^+ ^T cells [8,13,14], macrophages [15,16] and follicular dendritic cells [17]. The key anatomical viral reservoirs are the central nervous system (CNS), lymphoid organs [18] and the genitourinary tract [10,11]. The presence of HIV within the brain and CSF is directly associated with the development of a syndrome called HIV-associated dementia (HAD), which is characterized by a collection of cognitive, motor and behavioural symptoms [19]. In order to reduce the occurrence of HAD, therapeutic concentrations of antiviral drugs must reach the CNS. In support of this, it has been demonstrated that drugs which are able to reach the CSF, improve CNS function in HIV-infected individuals, as measured by psychomotor testing, a sensitive predictor of HAD [20,21]. Furthermore, sub-therapeutic levels of anti-HIV agents within the CNS may permit the evolution of drug resistant viral strains in the CSF, which have the potential to re-infect the periphery [11]. Several studies have provided evidence for the development of drug resistant HIV strains in the CSF independently of the plasma [22-24]. The presence of drug resistant variants of HIV is a major clinical concern as it is associated with therapy failure, disease progression and death. A clear understanding of drug distribution into these cellular and anatomical reservoirs is consequently of interest and could aid the rationale design of drug combinations to treat HIV infection more effectively. Entry of anti-HIV drugs into the CNS is partly limited by the blood-brain barrier (BBB) located at the cerebral capillary endothelium, and the blood-CSF barrier found at the choroid plexuses and the arachnoid membrane. This study focuses on the distribution of PMPA to the CNS by means of a brain/choroid plexus perfusion technique in anaesthetised guinea-pigs.

## Methods

### Brain perfusion technique

All the experiments were authorized under the Scientific Procedures Act (UK, 1986). Adult guinea-pigs (B & K, Grimston Hull, UK), n = 45, were anaesthetized with 0.32 mg/kg fentanyl and 10 mg/kg fluanisone (Hypnorm; Jansenn Animal Health, High Wycombe, UK) and 5 mg/kg midazolam (Hypnovel, Roche, Switzerland) intraperitoneally. They were heparinised with 25,000 units heparin sodium/ml, 1 ml/kg by the same route. The common carotid arteries were cannulated with fine silicon tubing connected to a perfusion pump as previously described [25]. The perfusion fluid consisted of an artificial plasma (NaCl 117.0 mM, KCl 4.7 mM, MgSO_4 _0.8 mM, NaHCO_3 _24.8 mM, KH_2_PO_4 _1.2 mM, CaCl_2_·6H_2_O 2.5 mM, D-glucose 10 mM, dextran MW 60 000–90 000 (MP Biochemicals, Aurora, OH, USA) 39 g/L and bovine serum albumin 1 g/L), which had been thoroughly oxygenated, debubbled and warmed to 37°C before passing into each carotid artery. With the start of perfusion, the jugular veins were sectioned and [^3^H]PMPA and D-[^14^C]mannitol dissolved in the artificial plasma could be infused into the inflowing perfusion medium via a slow drive syringe pump. Final concentrations in the perfusion medium were 6.5 nM for [^3^H]PMPA and 0.96 μM for D-[^14^C]mannitol. After varying perfusion times ranging from 2.5 to 30 min, a cisterna magna CSF sample was taken and the animal decapitated.

### Scintillation counting and capillary depletion analysis

Cerebrum, cerebellum, pituitary gland, lateral ventricle choroid plexuses, CSF and plasma samples were prepared for radioactive liquid scintillation counting by the addition of tissue solubiliser (Solvable; Packard Berkshire, UK) and liquid scintillation fluid (Lumasafe: Packard). Cerebrum samples were also taken for capillary depletion analysis by dextran density centrifugation as previously reported [25]. This method determines if a drug has actually crossed the BBB as it examines drug accumulation by the cerebral capillary endothelial cells and ensures any drug accumulation in the brain is not just a consequence of drug being trapped within the cerebral capillary endothelium. In brief, approximately 500 mg of cerebrum was homogenised in a glass homogeniser with 1.5 ml capillary depletion solution (HEPES 100 mM; NaCl 141 mM; KCl 4 mM; CaCl_2_·2H_2_O 2.8 mM; MgSO_4_·3H_2_O 1 mM; NaH_2_PO_4_·2H_2_O 1 mM; D-glucose 10 mM) before the addition of 2 ml dextran solution (26% w/v in water) and further homogenisation. Duplicate samples of this homogenate were taken, and the remainder was separated into two microcentrifuge tubes and centrifuged for 15 min (5400 g, 4°C). The resulting supernatant consisting of the brain parenchyma and the pellet rich in cerebral capillaries were separated, and together with the whole brain homogenate samples, prepared for liquid scintillation counting as described above.

### Self- and cross-inhibition experiments

Brain/choroid plexus perfusions were also carried out with the addition of 50 μM unlabelled PMPA, 100 μM azidodeoxythymidine (AZT), 100 μM 2',3'-didehydro-3'-deoxythymidine (D4T), 100 μM 2'3'-dideoxycytidine (ddC) or 100 μM abacavir in the artificial plasma. A further inhibitor study used 350 μM unlabelled probenecid, which is a broad inhibitor of organic anion transport, in the perfusion medium. All inhibitor studies had a perfusion time of 20 min.

### Expression of results

The amount of radioactivity in all the tissue samples was expressed as a percentage of the radioactivity in the plasma and termed uptake (%). When necessary, [^3^H]PMPA uptake was corrected for D-[^14^C]mannitol space by subtracting the D-[^14^C]mannitol uptake from the [^3^H]PMPA uptake.

### HPLC/radio detector analysis

The integrity of the tritium label to PMPA and the stability of PMPA in the artificial plasma were determined by HPLC/radio detector analysis. Samples were taken from the artificial plasma both before (arterial inflow) and after (venous outflow) it had passed through the cerebral circulation. Venous outflow samples were collected after the brains had been perfused for 5 and 25 min. The presence of intact [^3^H]PMPA in samples of the arterial inflow and venous outflow was determined by counter-ion reverse phase chromatography with radioactive flow detection (modified from [1]). Samples were prepared for analysis as follows. Venous outflow, collected after 5 or 25 min of perfusion, was centrifuged (3000 g, 5 min 4°C, Denley BR401 refrigerated centrifuge, Billingshurts, West Sussex, UK) and 75 μl of the supernatant was placed in a microcentrifuge tube, to which 75 μl acetonitrile was added before vortexing and centrifugation (13,000 g, 5 min, 4°C). The supernatant was then removed and diluted with HPLC grade water to achieve a final acetonitrile concentration of less than 10%. The sample was then lyophilised with N_2 _to a suitable volume for HPLC analysis. Samples of the arterial inflow were diluted 1:1 with acetonitrile and prepared for HPLC analysis in an identical manner. The HPLC system comprised of HG-1580 high pressure, high performance gradient HPLC solvent delivery system, AS-1555-10 cooled autosampler and UV-1575 UV/Vis detector (Jasco, Essex, UK) linked to a Flow Scintillation Analyser (500 TR series, Packard, Berks, UK). A Luna 3 μm C18 (2) column, 150 × 4.6 mm, equipped with an ODS Security Guard cartridge (Phenomenex, Cheshire, UK) was utilised. Two mobile phases were used: phase A: 5% acetonitrile in HPLC grade water with 20 mM potassium phosphate and 5 mM tetrabutylammonium dihydrogen phosphate and phase B: 65% acetonitrile in HPLC grade water with 20 mM potassium phosphate and 5 mM tetrabutylammonium dihydrogen phosphate. The gradient was linear from 100% phase A to 100% phase B over 15 min. The flow rate was 1 ml/min and the injection volume was 100 μl. This was mixed with Ultima-Flo M scintillation fluid (Packard) and passed through the Flow Scintillation Analyser for radioactive analysis. The data was acquired and stored with a Flo-One data acquisition system (Packard).

### Octanol-saline partition coefficient

An octanol-saline partition coefficient was determined for [^3^H]PMPA. For this 0.75 ml phosphate buffered saline (pH 7.4) containing [^3^H]PMPA was added to a microcentrifuge tube with 0.75 ml octanol and vortexed. This was then centrifuged for 5 min (1000 g 4°C) and triplicate 100 μl samples of the upper phase (octanol) and lower phase (saline) were taken for radioactive scintillation counting. The octanol-saline partition coefficient (mean radioactivity in octanol samples/mean radioactivity in saline samples) of [^3^H]PMPA was determined in triplicate and reported as the mean ± the standard error of the mean.

### Chemicals

(9-[9(R)-2-(phosphonomethoxy)propyl]adenine, [adenine-2,8-^3^H] ([^3^H]PMPA, specific activity 12.5 Ci/mmol), D-[^14^C]mannitol (specific activity 53 mCi/mmol) and unlabelled PMPA (MW 287.2) were purchased from Moravek Biochemicals Inc (California, USA) and stored at -20°C, as recommended by the manufacturers. All other chemicals were bought from Sigma Chemical Company (Dorset, UK).

## Results

### Entry into brain and pituitary

**Figure 2:**
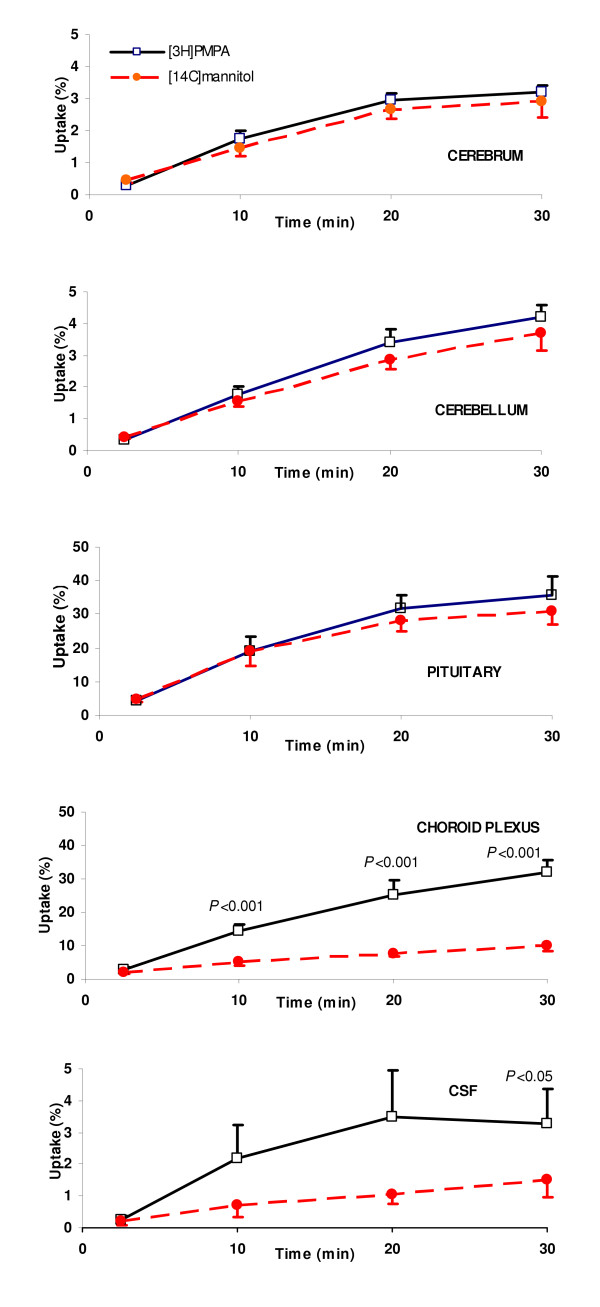
The uptake of [^3^H]PMPA and D-[^14^C]mannitol into the different CNS regions measured against perfusion time. Data at each time point represents mean ± S.E.M., n = 3–8. When the uptake of [^3^H]PMPA was significantly greater than that for D-[^14^C]mannitol the level of significance is indicated. Elsewhere there was no significant difference between [^3^H]PMPA and D-[^14^C]mannitol in that tissue compartment at that time point.

The uptake data for [^3^H]PMPA and D-[^14^C]mannitol measured over various perfusion times into the different regions of the CNS is illustrated in Fig. 2. The uptake of radiolabeled PMPA into the cerebrum ranged from 0.3 ± 0.1 ml/100 g at 2.5 min to 3.2 ± 0.2 ml/100 g at 30 min. The uptake of the vascular space marker molecule, D-[^14^C]mannitol, was 0.5 ± 0.1 ml/100 g at 2.5 min and 2.9 ± 0.5 ml/100 g at 30 min. The uptake of [^3^H]PMPA was not significantly greater than that measured for D-[^14^C]mannitol into this region at any time point (paired Student's *t*-test). The uptake of [^3^H]PMPA and D-[^14^C]mannitol into the cerebellum at 2.5 min was 0.3 ± 0.1 and 0.4 ± 0.1 ml/100 g, respectively, and at 30 min was 4.2 ± 0.4 and 3.7 ± 0.5 ml/100 g, respectively. The [^3^H]PMPA and D-[^14^C]mannitol cerebellum values were not significantly different from each other at any time point (paired Student's *t*-test). Similarly, the uptake of radiolabeled PMPA and mannitol into the pituitary gland were not significantly different from each other at any time point (paired Student's *t*-test).

### Entry into choroid plexus and CSF

The accumulation of [^3^H]PMPA within the choroid plexus tissue was significantly greater than D-[^14^C]mannitol at 10, 20 and 30 min (Fig. 2, *P *< 0.001, paired Student's *t*-test or Wilcoxon signed rank test, as appropriate). The movement of [^3^H]PMPA into the CSF was also significantly greater than that measured for D-[^14^C]mannitol at 30 min (*P *< 0.05, paired Student's *t*-test), but not at any other time point.

### Capillary depletion analysis

**Figure 3:**
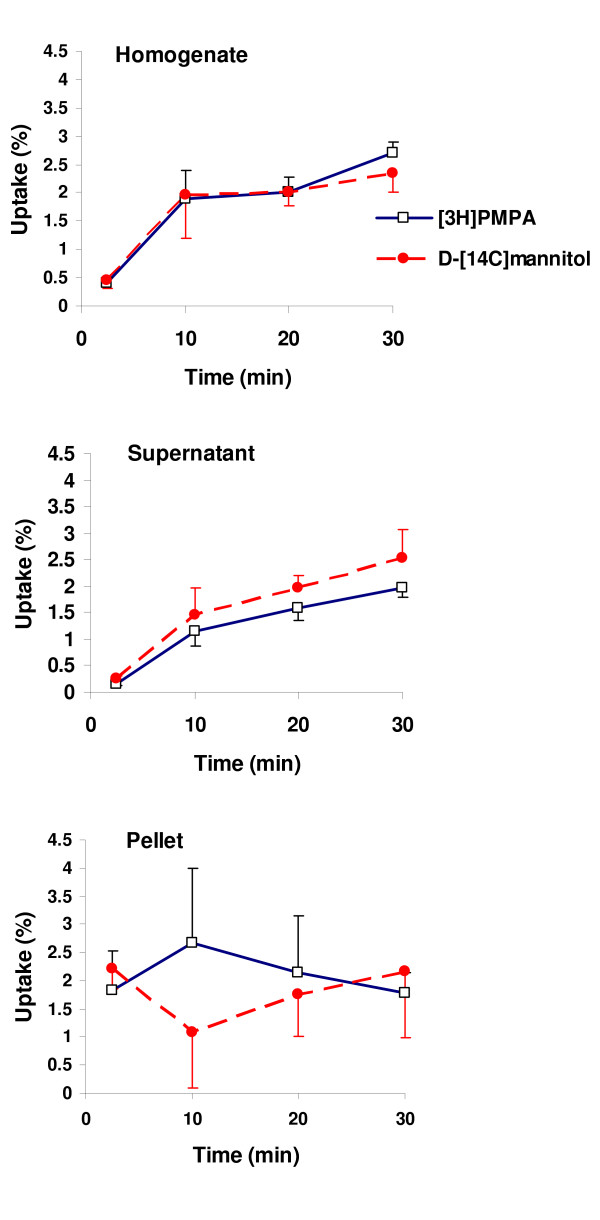
Capillary depletion analysis of [^3^H]PMPA and D-[^14^C]mannitol perfused cerebrum samples. Each point represents mean ± S.E.M., n = 3–6. There were no significant differences between the distribution of [^3^H]PMPA and D-[^14^C]mannitol in the whole brain homogenate, the brain parenchyma (supernatant) or the cerebral capillary endothelial cell enriched pellet at any time point. Accumulation in the pellet and supernatant fractions does not equal the uptake measured in the homogenate because all the data was standardized by weight. It is estimated that the cerebral capillary endothelial cell component only represents approximately 0.1% of the whole brain homogenate.

Capillary depletion analysis of cerebrum samples from perfused brains showed that there was no significant difference between [^3^H]PMPA and D-[^14^C]mannitol in the whole tissue homogenate, parenchyma (supernatant) or the cerebral capillary cell enriched pellet at any of the time points (Fig. 3).

### Entry in the presence of inhibitors

**Figure 4:**
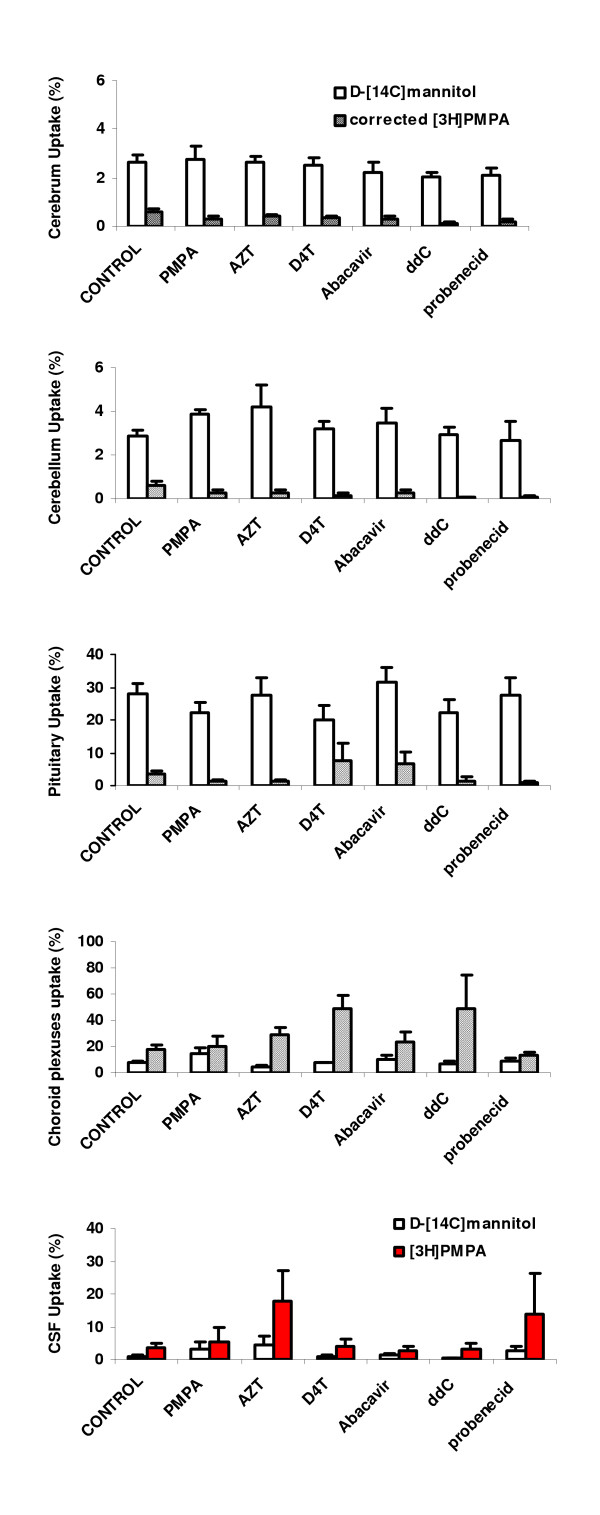
The CNS uptake of [^3^H]PMPA in the presence of unlabelled PMPA, antiretroviral drugs and probenecid at a perfusion time of 20 min. The cerebrum, cerebellum, pituitary and choroid plexuses values of [^3^H]PMPA were corrected for vascular space or extracellular space, as measured by D-[^14^C]mannitol. The CSF uptake of [^3^H]PMPA is uncorrected for D-[^14^C]mannitol, as in this tissue it represents a paracellular permeability marker molecule. The uptake of D-[^14^C]mannitol in the presence of any of the additional drugs was not significantly different to that achieved in their absence (control) and demonstrates that at these concentrations of unlabelled drugs the integrity of the barriers is maintained. The presence of these additional drugs also did not significantly affect the distribution of [^3^H]PMPA. Each time point represents mean ± S.E.M., n = 3–7.

There was no difference in the movement of [^3^H]PMPA into the cerebrum, cerebellum, pituitary, choroid plexuses or CSF in the presence of the tested anti-HIV drugs and the broad spectrum efflux inhibitor, probenecid (one-way ANOVA or Kruskal-Wallis one-way ANOVA on ranks, as appropriate, Fig. 4). There was also no significant difference between the values achieved for the distribution of D-[^14^C]mannitol into any of the CNS regions in the presence of the tested drugs, data not shown.

### Octanol-saline partition coefficient

The octanol-saline partition coefficient for [^3^H]PMPA was 0.0134 ± 0.00003 (n = 3).

### Stability of [^3^H]PMPA in plasma

**Figure 5:**
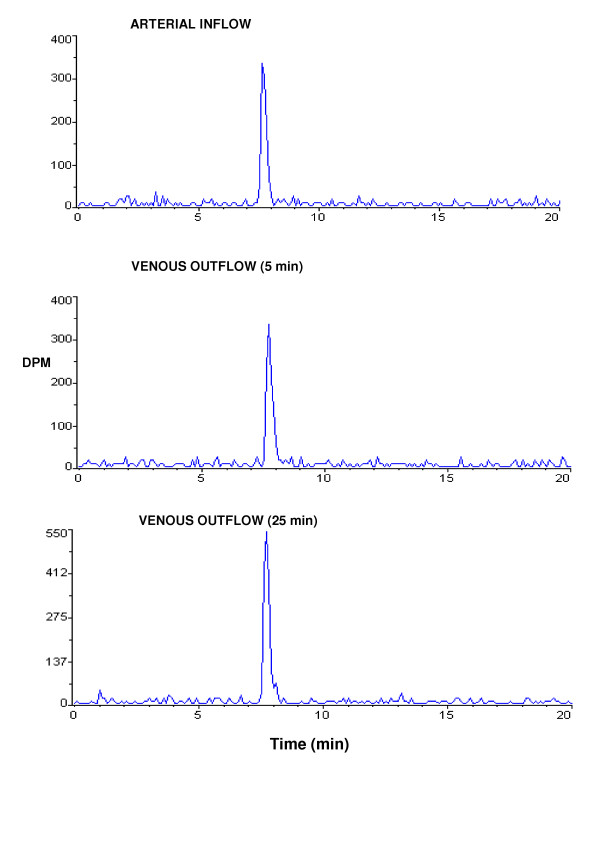
HPLC-radio detector analysis of arterial inflow and venous outflow samples taken after 5 and 25 minutes of perfusion with [^3^H]PMPA showing that the majority of the radioactivity present in the cerebral circulation was intact radiolabeled PMPA.

HPLC/radio detector analysis was performed on the arterial inflow and venous outflow at 5 and 25 min perfusion times taken from [^3^H]PMPA brain perfusion experiments. The majority of the radioactivities in these plasma samples were eluted at approximately 7.7 min (Fig. 5). This retention time is consistent with that for the PMPA standard and confirms that the majority of the radioactivity present in the cerebral circulation was intact radiolabeled PMPA.

## Discussion

### Uptake of [^3^H]PMPA into CNS tissues

PMPA was used in this study as it is the molecular form of the nucleotide reverse transcriptase inhibitor (NtRTI) in the circulation and thus would be the form exposed to the luminal surfaces of the blood-brain and blood-CSF interfaces. In this present study the uptake of intact [^3^H]PMPA into the cerebrum, cerebellum and cerebral capillary endothelial cell enriched pellet was no greater than that measured for the marker molecule, D-[^14^C]mannitol (Figs. 2, 3 and 4). Radiolabeled mannitol transport across the BBB has been investigated in an earlier study where rats were dosed intravenously and the brain uptake of D-[^3^H]mannitol was corrected for vascular space using radiolabeled indium as a marker of blood plasma space [26]. Low levels of D-[^3^H]mannitol were measured in the brain parenchyma with values being 1.2% of plasma concentrations after 10 min, rising to 3.3% after 4 h. Work by Sisson and Oldendorf demonstrated that the initial uptake (0–10 min) of D-[^3^H]mannitol into rat brain is rapid, and this is the result of mannitol entering a small rapidly equilibrating brain space, suggested to be the area between the capillary endothelium and the neurological lining of the capillary, or the capillary endothelium itself [26]. This rapid uptake is followed by slow steady uptake into the brain compartment. Hence within the timescale of the experiments described in this current study, D-[^14^C]mannitol uptake into the brain mainly represents vascular space within this tissue, plus slow paracellular diffusion between the BBB endothelial cells. Thus the results show that the integrity of the brain barriers was maintained since the D-[^14^C]mannitol values were similar to those previously reported by Sisson and Oldendorf for the rat [26] and by our own research group in the guinea pig [27]. Hence, D-[^14^C]mannitol uptake provides a baseline to which PMPA uptake into the brain can be compared. It is important to note that PMPA is not highly bound to proteins with 99% remaining unbound in the plasma [6], and this is also indicated in this present study when an excess of unlabelled PMPA failed to change the CNS distribution of [^3^H]PMPA. This fact, together with the uptake data indicates that [^3^H]PMPA has a very limited ability to cross the BBB. This is certainly linked to the hydrophilic nature of PMPA as measured by its low octanol-saline partition coefficient of 0.013, which is higher than the coefficient previously measured for D-[^14^C]mannitol (0.002 ± 0.0004 [25]), but lower than other non-BBB penetrating molecules such as urea (0.03 [28]). Another interesting observation is that the distribution of [^3^H]PMPA into the pituitary gland, although much greater than into brain tissue, was not significantly different to that observed for D-[^14^C]mannitol. The pituitary gland consists of two distinct parts, the posterior and the anterior pituitary. The neural lobe of the pituitary (part of the posterior pituitary) lies outside the BBB and thus the capillaries in this region are more permeable than the BBB capillaries, allowing the free exchange of circulating substances between the blood and pituitary gland [29]. In the pituitary gland, D-[^14^C]mannitol would be able to cross the capillary endothelium freely by passing between the cells, and hence act like a paracellular permeability marker molecule. The lack of a difference between this marker molecule and the test NtRTI in the pituitary gland would suggest that PMPA, acts like D-[^14^C]mannitol, and is also crossing the capillary endothelium paracellularly in the pituitary gland. In contrast to this, the choroid plexus tissue had a significantly greater accumulation of [^3^H]PMPA compared to the simultaneously measured D-[^14^C]mannitol. The nature of the choroid plexus capillaries allows mannitol to penetrate into the stromal space, however, mannitol does not gain access to the cellular compartment of the choroid plexus [30]. Overall, the results of this present study would suggest that [^3^H]PMPA is able to cross the cell membranes of the choroid plexus. It is not possible to determine if the PMPA is accumulating in the choroid plexus endothelium or the epithelium or in both, but together with the data illustrated in Fig. 3 and the low lipophilicity of PMPA, these results suggest that a transport system may be involved in the movement of PMPA into the choroid plexus. Another interesting observation is that [^3^H]PMPA does reach the CSF in the cisterna magna. As there was no detectable movement of [^3^H]PMPA across the BBB, this would suggest that it has come from movement across the blood-CSF barrier, principally the choroid plexuses. The tight junctions at the choroid plexuses are considered to be 'leakier' and hence more permeable, than the capillary endothelium, due to the differences in the continuity of the tight junctions between these two vascular-CNS interfaces [31,32]. Thus drugs have previously been seen to cross the blood-CSF barrier, but not the BBB [33]. The higher movement of [^3^H]PMPA into the CSF compared with D-[^14^C]mannitol after 30 min of vascular perfusion could also indicate that [^3^H]PMPA is crossing transcellularly, in addition to paracellularly. This is also confirmed by the presence of [^3^H]PMPA within the cells of the choroid plexus tissue as measured by its higher accumulation than D-[^14^C]mannitol in this compartment. Overall this would indicate that PMPA would have rapid access to the HIV infected perivascular and meningeal macrophages [34,35], but it is unlikely to be able to eradicate virus in the microglia, which together with the perivascular macrophages are thought to be the major populations of infected cells within the CNS [36-39].

### Possible involvement of transporters

Drug movement across the blood-brain and blood-CSF barriers can occur by diffusion or through the action of transport systems. Several transport systems exist at the brain-barriers and can facilitate the movement of molecules into and out of the CNS. As there was differential handling of [^3^H]PMPA and D-[^14^C]mannitol at the level of the choroid plexuses compared with the cerebral capillary endothelium, some form of transporter involvement is suggested. This is likely to be at the choroid plexus due to the hydrophilic nature of PMPA and the lack of any significant accumulation of PMPA by the cerebral capillary endothelial cell enriched pellet. Further studies investigated if we could detect any saturable transporter for PMPA using an excess concentration (50 μM) of unlabelled PMPA. This concentration was chosen based on the free tenofovir plasma C_max _of 0.8–1.3 μM measured after a therapeutic dose of the tenofovir pro-drug had been administered to HIV patients [3]. However, none of the tissues we examined, including the choroid plexus, indicated the presence of a high affinity transporter for PMPA. It is known that [^3^H]PMPA is efficiently transported by the human renal organic anion transporter (OAT1) [40]. PMPA also shows a moderate inhibition of renal hOAT3 with a half-saturation transport constant (K_m_) of 3.3 mM [41]. Thus hOAT3 is a low affinity transporter for PMPA, where as hOAT1 appears to a function as a high-affinity transporter with a K_m _of 34 μM. OAT1 has been detected in the brains of humans, rats and mice, and OAT1 is expressed in the choroid plexus of mice and rats [42] albeit at low levels [43,44], where it is understood to be located on the apical membrane of the epithelial cells and can function to efflux organic anions from the CSF [42,45]. Although there is no direct evidence for the expression of OAT1 at the BBB, it is suggested that the observed efflux of certain substances known to be OAT1 substrates (including AZT and benzylpenicillin) from brain to blood, indicates that the presence of OAT1 at the BBB is likely [46]. OAT3 is expressed in the human and rat CNS [47,48]. In the brain, OAT3 is localized at the apical membrane of the choroid plexus [42,49] and at the abluminal membrane of capillary endothelial cells [50]. PMPA has also been shown to be a substrate for multidrug resistance protein 2 (MRP2) in cell lines transfected with the transporters [41]. Probenecid, as used in this study, is a broad inhibitor of organic anion transport, and is known to inhibit the members of the OAT family, as well as the organic anion transporter polypeptide (OATP/oatp) and MRP families. However, there was no detectable interaction of [^3^H]PMPA with these transporters as determined by the absence of a significant affect on CNS distribution when probenecid was present in the perfusion medium.

An interesting aspect of the use of transporters by anti-HIV drugs is that certain combinations of anti-HIV drugs could interact for transport at the brain barriers. For example, the concentration of 2'3'-dideoxyinosine (ddI) in the guinea-pig choroid plexus was altered in the presence of the other NRTIs, abacavir, (-) -β-L-2'3'-dideoxy-3'-thiacytidine (3TC) and AZT [51]. In addition, ritonavir accumulation by the perfused choroid plexus was significantly reduced by nevirapine and abacavir [27]. This could ultimately result in sub-therapeutic concentrations of drugs within the brain/choroid plexus/CSF, which would allow viral replication to continue and drug resistant strains of HIV to be selected. A clear understanding of the use of transport mechanisms by the anti-HIV drugs could clearly aid in the design of more effective drug regimens. For this reason, a series of cross-competition studies were also carried out with other anti-HIV drugs in the artificial plasma. There was no significant change in the CNS accumulation of radiolabeled PMPA in the presence of AZT, D4T, abacavir or ddC. Previous studies have also found no evidence for an antagonistic pharmacokinetic/pharmacodynamic interaction between PMPA and abacavir [52-55]. However, unexpected early virological failure has been seen with the once-daily triple-NRTI combinations of ddI/3TC/PMPA and abacavir/3TC/PMPA [56], but this is not seen when these agents are also used with a protease inhibitor [6,57]. Overall these results of drug combination experiments suggest that PMPA should function effectively as part of many anti-HIV combination therapeutic regimens.

## Conclusion

PMPA has been shown to cross the blood-CSF barrier and reach the CSF, but it cannot cross the BBB to reach deep brain sites. Overall this would suggest that it should only be included in a regimen with anti-HIV drugs already proven in their capacity to prevent or alleviate the neurological problems associated with HIV infection or AIDS.

## List of abbreviations

HIV: human immunodeficiency virus, BBB: blood-brain barrier, CNS: central nervous system, HAD: HIV-1-associated dementia, PMPA: 9-[9(R)-2-phosphononomethoxy)propyl]adenine, NtRTI: nucleotide reverse transcriptase inhibitor, HAART: Highly active anti-retroviral therapy, AZT: azidodeoxythymidine, D4T: 2',3'-didehydro-3'-deoxythymidine, ddC: 2'3'-dideoxycytidine, OAT: organic anion transporter, ddI: 2'3'-dideoxyinosine, OATP: organic anion transporter polypeptide, 3TC: (-) -β-L-2'3'-dideoxy-3'-thiacytidine, MRP: multidrug resistance protein.

## Declaration of competing interests

The author(s) declare that they have no competing interests.

## Authors' contributions

CA carried out the majority of the brain perfusion experiments. JEG carried out all the HPLC analyses and some of the brain perfusion experiments and data analyses. SAT conceived the study, designed the experimental plan, carried out the majority of the data and statistical analyses and wrote the majority of the manuscript. All authors read and approved the final manuscript.
